# A predictive model for the critical shoulder angle based on a three-dimensional analysis of scapular angular and linear morphometrics

**DOI:** 10.1186/s12891-022-05920-7

**Published:** 2022-11-22

**Authors:** Geoffrey C. S. Smith, Peter Geelan-Small, Michael Sawang

**Affiliations:** 1 Faculty of Medicine, University of New South Wales, Sydney, Australia; 2 Department of Orthopaedics, St George Hospital, Suite 201, Level 2, 131 Princes Highway, Kogarah, Sydney, NSW 2217 Australia; 3 St George and Sutherland Centre for Clinical Orthopaedic Research, Sydney, Australia; 4 Mark Wainwright Analytical Centre, Stats Central, University of New South Wales, Sydney, Australia

**Keywords:** Critical shoulder angle, Glenoid inclination, Rotator cuff tear, Acromial morphology, Acromioplasty

## Abstract

**Background:**

The purpose of this study was to define the features of scapular morphology that are associated with changes in the critical shoulder angle (CSA) by developing the best predictive model for the CSA based on multiple potential explanatory variables, using a completely 3D assessment.

**Methods:**

3D meshes were created from CT DICOMs using InVesalius (Vers 3.1.1, RTI [Renato Archer Information Technology Centre], Brazil) and Meshmixer (3.4.35, Autodesk Inc., San Rafael, CA). The analysis included 17 potential angular, weighted linear and area measurements. The correlation of the explanatory variables with the CSA was investigated with the Pearson’s correlation coefficient. Using multivariable linear regression, the approach for predictive model-building was leave-one-out cross-validation and best subset selection.

**Results:**

Fifty-three meshes were analysed. Glenoid inclination (GI) and coronal plane angulation of the acromion (CPAA) [Pearson’s r: 0.535; -0.502] correlated best with CSA. The best model (adjusted R-squared value 0.67) for CSA prediction contained 10 explanatory variables including glenoid, scapular spine and acromial factors. CPAA and GI were the most important based on their distribution, estimate of coefficients and loss in predictive power if removed.

**Conclusions:**

The relationship between scapular morphology and CSA is more complex than the concept of it being dictated solely by GI and acromial horizontal offset and includes glenoid, scapular spine and acromial factors of which CPAA and GI are most important. A further investigation in a closely defined cohort with rotator cuff tears is required before drawing any clinical conclusions about the role of surgical modification of scapular morphology.

**Level of evidence:**

Level 4 retrospective observational cohort study with no comparison group.

## Background

**Fig. 1:**
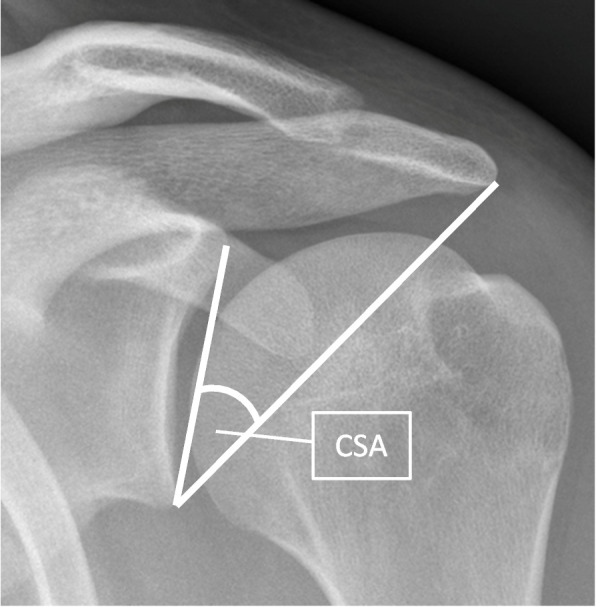
Measurement of the critical shoulder angle using an anteroposterior radiograph of the shoulder. The angle is between a line connecting the inferior with the superior border of the glenoid fossa and another connecting the inferior border of the glenoid with the most inferolateral point of the acromion

The critical shoulder angle (CSA) is the angle between a line connecting the superior and inferior aspects of the glenoid fossa and another line connecting the inferior aspect of the glenoid with the most inferolateral point of the acromion on a plain anteroposterior radiograph [32] (Fig. 1). Variations in the CSA are associated with rotator cuff (RC) tears (CSA > 35°) and primary glenohumeral osteoarthritis (GHOA) (CSA < 30°), however, a temporal causal relationship has not been established [2, 4, 7, 8, 12, 19, 31–33, 36, 37, 39, 41]. Proven aetiological factors in the development of RC tears and primary GHOA include a genetic predisposition, degenerative processes and pathological loading applied through occupational, recreational, or accidental exposure [11, 14, 20, 27, 48]. The common factor of pathological loading in the development of both RC tears and primary GHOA is potentially explained by a mechanism through which scapular morphology may influence the size and composition of the non-rotational force generated by deltoid contraction. This could be affected by the location of the lateral acromion relative to the centre of rotation of the shoulder and the direction of the deltoid non-rotational force vector relative to the glenoid face. High CSA values are associated with greater than normal supraspinatus forces which are required to maintain glenohumeral stability in the face of a higher shear component, and low CSA values with a higher compressive component of the joint reaction force [18, 47].

**Fig. 2:**
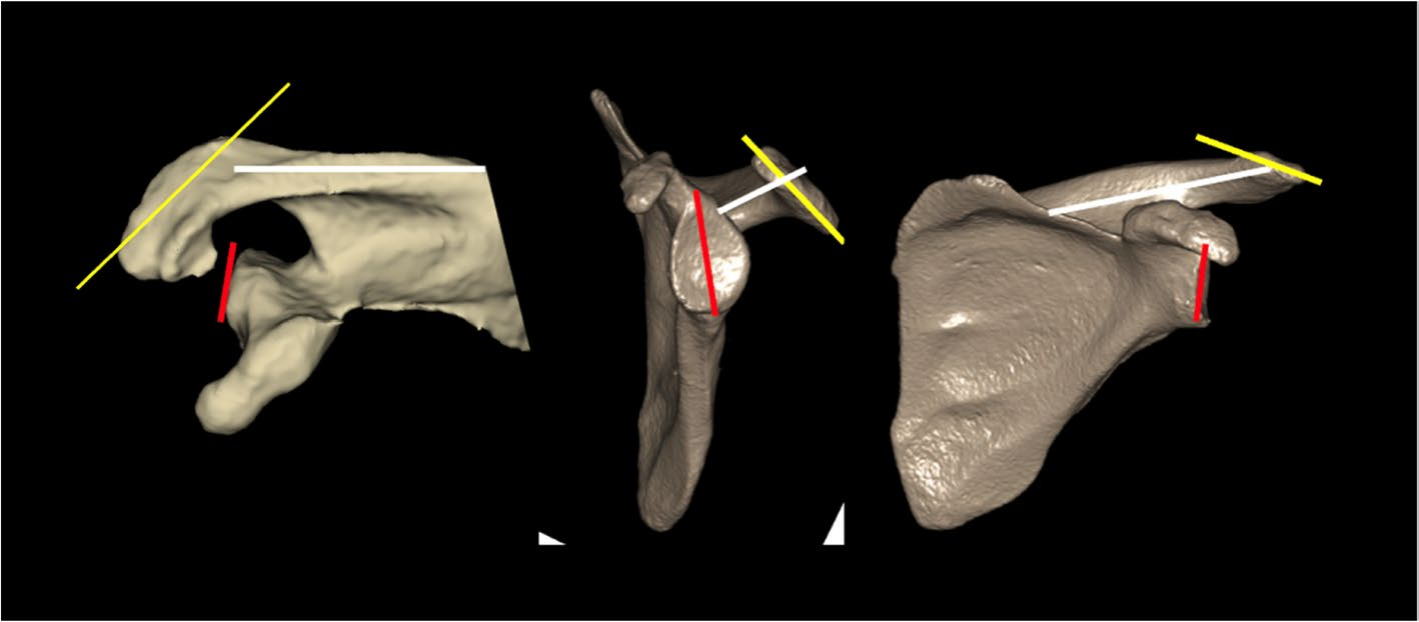
Illustration of the potential location of the 3D scapular variations underlying the 2D CSA value which may be related to the orientation of geometry of the glenoid (red), scapular spine (white) or acromion (yellow)

However, the CSA is a 2D surrogate marker of 3D scapular morphology that gives no information about its underlying scapular variations which may lie in the glenoid, scapular spine or acromion (Fig. 2) [9]. An investigation of the interindividual scapular morphological variations that underly different CSA values is therefore warranted [9].

**Fig. 3:**
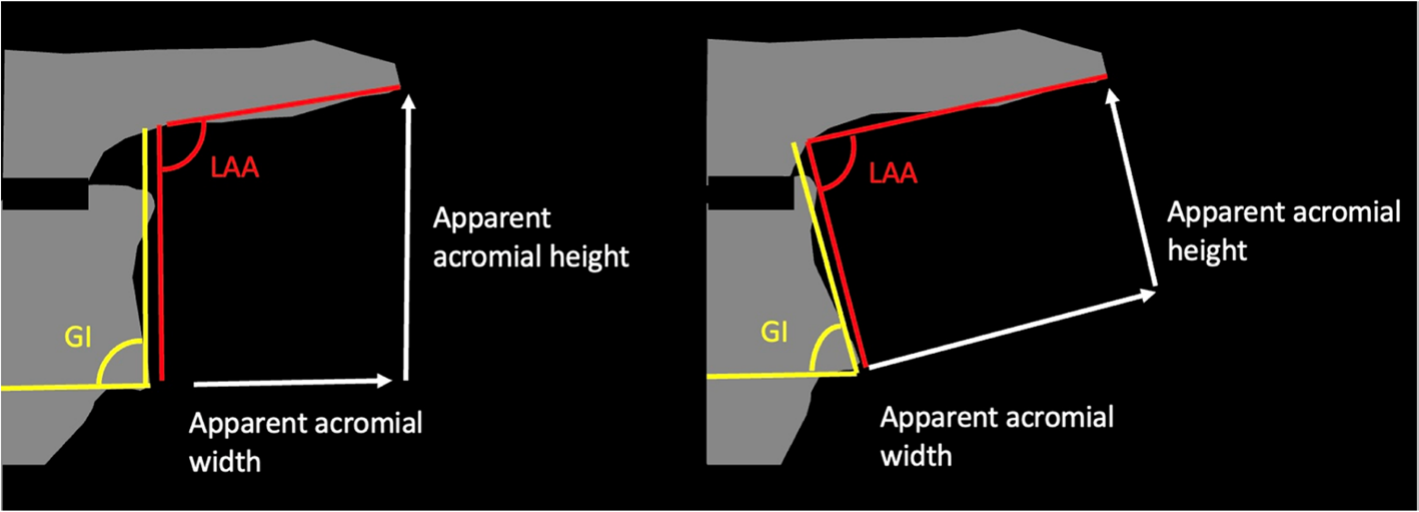
Illustration of the effect of glenoid morphological variation on non-glenoid morphological linear and angular measurements. An increase in glenoid inclination (GI) results in a decrease in the lateral acromial angle (LAA), increase in the apparent acromial width and a decrease in the apparent acromial height despite of no changes in the scapular spine or acromion

Several previous studies of scapular morphology have used 2D angular measurements and ratios of linear measures using plain radiographs, 2D CT or 2D measurement of 3D CT images [3–5, 16, 26, 33–35, 38, 43] However, the scapula is a complex 3D structure, and these 2D techniques may not adequately differentiate between morphological variations that are relevant to the CSA value [28]. In particular, the glenoid face is commonly used as a reference axis for angular and linear measurements [5, 33, 34, 38, 43]. If this is the case, the resultant non-glenoid measurements of geometry and orientation are affected by glenoid variations and their values are potentially misleading (Fig. 3).

Geometric morphometric analysis and statistical shape modelling (SSM) are alternative advanced approaches that allow a 3D assessment of morphometry [28, 44, 45]. Here, landmarks are assigned to surface models and subsequent analysis allows for the identification of those changes that explain the greatest shape variation [28]. These techniques have been applied to the scapula, demonstrating that progressive superior glenoid inclination combined with cranial orientation of the scapular spine and/or narrowing of the subacromial space are associated with RC tears [28]. However, these differences do not identify the exact location of the underlying variation. Furthermore, the correlation of models generated by SSM with previously described 2D angular measurements that have been linked with RC tears and GHOA (such as the CSA) require 2D measurements of the generated models, negating the benefits of a 3D analysis [44, 45].

Alternatively, the definition of bony landmarks that allow for the decomposition of the scapula into subparts and their subsequent 3D morphometry (by measurement of their geometry [length, width, and height] and orientation [angular displacement around three mutually perpendicular axes away from a reference frame] could allow an assessment of their individual variations [22]. While this approach has been applied to the scapula this has not been done with specific reference to the CSA value, and only with regards to orientation and not geometry [22].

Some landmarks for morphological assessment of the scapula have been described but these are not suitable for the decomposition of the scapula into subparts and therefore additional alternative landmarks would be required for this approach [10]. The scapular (x) axis, and perpendicular axes running anteroposteriorly (y axis) and superoinferiorly (z axis) through the centre of the glenoid can be used as reference axes to define the orientation of the scapular subparts away the scapular plane, which is a suitable reference frame [15].

**Fig. 4:**
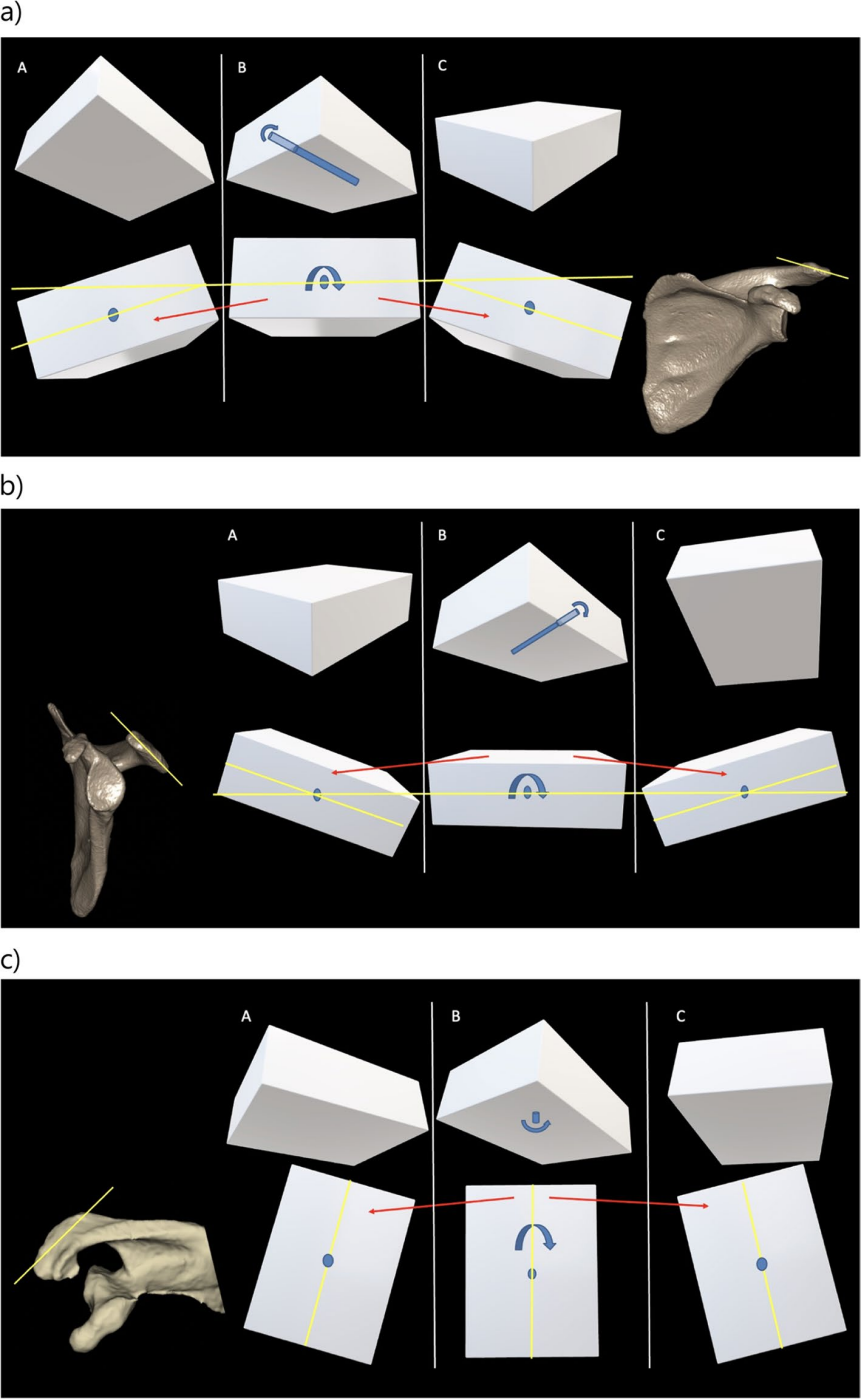
**a** Schematic diagram of changes in acromial coronal plane orientation: A, low coronal plane angulation of the acromion (CPAA); B, neutral CPAA; C, high CPAA. **b** Schematic diagram of changes in acromial sagittal plane orientation: A, low sagittal plane angulation of the acromion (SPAA); B, neutral SPAA; C, high SPAA. **c** Schematic diagram of changes in acromial axial plane orientation: A, high axial plane angulation of the acromion (APAA); B, neutral APAA; C, low APAA

Once suitably decomposed, the orientation of the glenoid, acromion and scapular spine can be described according to the axis that is most closely aligned to its own long axis [22]. The nomenclature for the orientation of the scapula subparts has not been established, but regarding acromial orientation, because its long axis is most closely aligned to the sagittal plane, acromial rotation about the x axis can be termed sagittal plane acromial angulation (SPAA); rotation about the y axis can be termed coronal plane acromial angulation (CPAA); and rotation about the z axis can be termed axial plane acromial angulation (APAA) (Fig. 4). Regarding the scapular spine orientation, as its long axis is closest to the coronal plane, rotation about the x axis can be termed sagittal plane scapular spine angulation (SPSSA); rotation about the y axis can be termed coronal plane scapular spine angulation (CPSSA); and rotation about the z axis can be termed axial plane scapular spine angulation (APSSA). The lateral part of the acromion serves as a point of origin for the deltoid which is important in the biomechanical concept linking the CSA to RC tears and primary GHOA [18, 40, 46]. The assessment of the geometry and orientation of the lateral acromion may therefore be more relevant than that of the entire acromion.

The use of the scapula is already established as a reference for the description of the orientation of the glenoid where glenoid version (GV) is the angulation of the glenoid relative to the scapula in the axial plane and glenoid inclination (GI) is the tilt of the glenoid relative to the scapula in the coronal plane [13, 16, 21, 30].

Finally, in a 3D assessment, the scapular plane could also potentially be used to improve the CSA measurement technique. The described 2D measurement of the CSA is affected by scapular positioning relative to the imaging plane, with accurate measurement requiring a near perfect overlap of the anterior and posterior glenoid rims [42]. This is difficult to achieve, resulting in many radiographs being unsuitable for accurate CSA measurement [39]. The use of a 3D image that is positioned perpendicular to the scapular plane (rather than the glenoid plane) is an alternative that would standardise scapular positioning for CSA measurement without relying on overlap of the glenoid rims.

The purpose of this study was to define the features of scapular morphology that are associated with changes in the CSA by developing the best predictive model for the CSA based on multiple potential explanatory variables, using a completely 3D assessment which employs the scapular plane as a reference plane and the scapular axis (and two other perpendicular axes) as a reference axis and incorporates the aforementioned comprehensive nomenclature for the description of the scapular variances.

## Methods

Ethics approval was obtained: University of New South Wales Human Research Ethics Committee HC180247. This was a retrospective observational cohort study of a diagnostic test. The clinical records of a single surgeon (GS) were searched over a 6-year period for those patients who underwent CT scan of the shoulder. During this time CT was performed routinely for all patients undergoing arthroplasty (anatomic and reverse), glenohumeral instability and some fractures. Exclusion criteria were: insufficient resolution (slice thickness > 1 mm); gross glenoid or acromial deformity (eg glenoid, scapular spine or acromial fracture, glenoid bone loss); artefact (beam hardening, CT arthrography); incomplete capturing of the entire scapula. The digital imaging and communications in medicine (DICOM) data from the included CT scans were imported into InVesalius (Vers 3.1.1, RTI [Renato Archer Information Technology Centre], Brazil), which was used to segment bone from the CT raw data and to create 3D meshes. The meshes were then imported into Meshmixer (3.4.35, Autodesk Inc., San Rafael, CA) for cleaning and analysis. All angular measurements were acquired via an orthographic view, eliminating any angular distortion which would otherwise arise from changes in perspective.

### 3D analysis principles

**Fig. 5:**
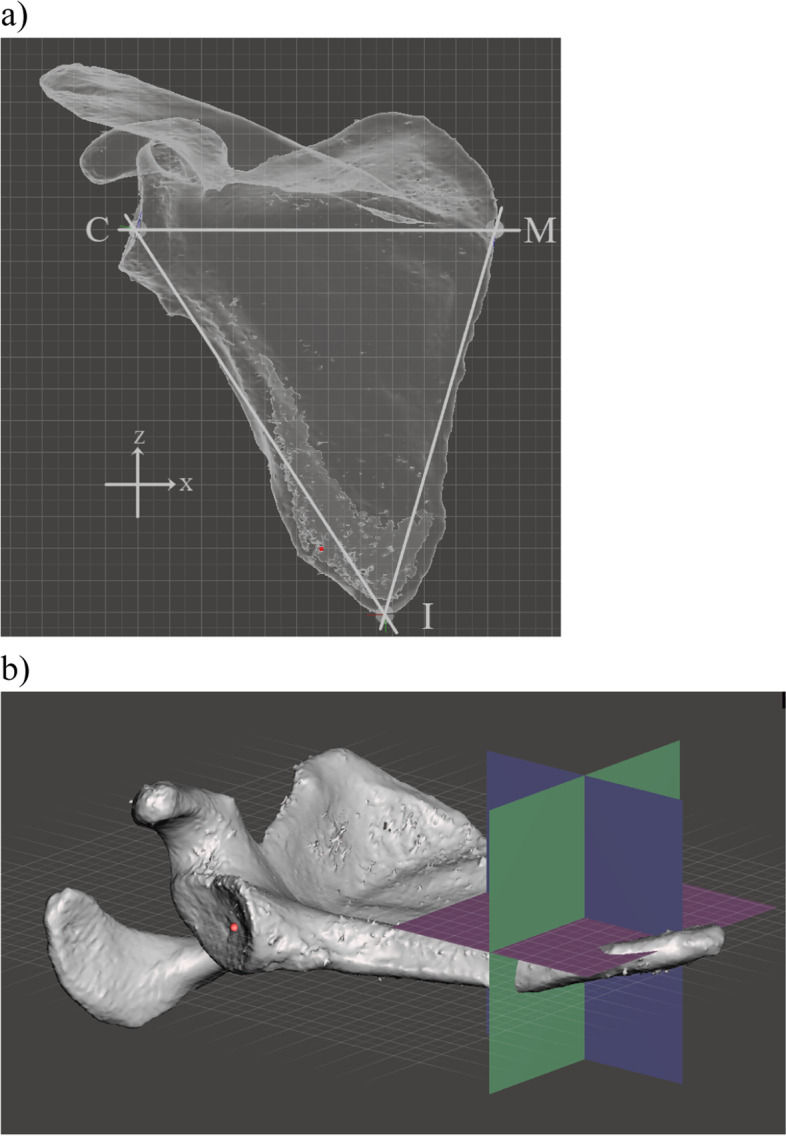
**a** The reference axes: the mesh has been orientated to the baseline position (perpendicular to the scapular plane, which is marked with a grid). The glenoid centre (point C, defined by the centre of a sphere of best fit based on the inferior glenoid and the anterior and posterior glenoid rims; the junction of spine of the scapula and the medial border of the scapula (point M); and the most distal point of the inferior scapular angle (point I) have been marked (points M and I were identified using multiple viewing perspectives). **b** The reference planes: Scapular plane (or x plane, purple), y plane (green) and z plane (blue). The scapular plane is marked by a grid. The ‘world origin point’ has been placed in the centre of the glenoid (red dot)

**Fig. 6:**
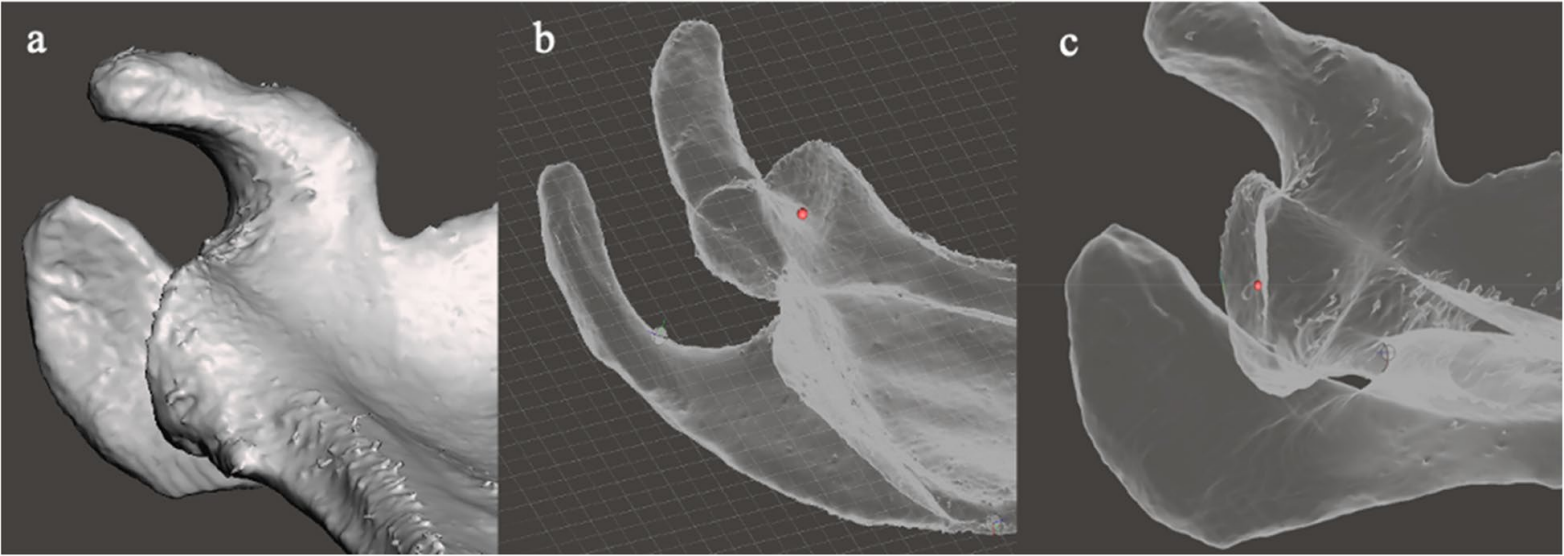
3D mesh images demonstrating: **a** undersurface acromion viewing plane. **b** posterolateral acromion viewing plane. The scapular plane is marked with a visible grid. The ‘world origin point’ has been placed in the centre of the glenoid (red dot). The location of the ‘acromiospinal point’ in this view has been marked with a spherical ‘pivot’. **c** use of a gridline to re-orientate the image. The mesh has been re-orientated to the axial viewing plane by ensuring that the x plane grid appears as a line

The ‘world origin point’ is the 0 point in the x, y and z axes. ‘Pivots’ were used to mark a point in 3D space relative to the ‘world origin point’.Pivots can also be used to define axes and planes (an axis by two pivots; a plane by three colinear pivots). Analysis initially involved the definition of the location of several key landmarks that were marked with pivots which were then used to generate reference axes and planes. The key landmarks were: the centre of the glenoid (point C); the junction of the spine of the scapula and the medial border of the scapula (point M); the inferior angle of the scapula (point I), which were selected as they have previously been used to define the scapular plane (Fig. 5a) [15]. The x axis was defined by the line CM. The y-axis is perpendicular to the x axis and runs anteroposteriorly through point C. The z-axis is perpendicular to the x axis running superoinferiorly through point C (Fig. 5b). The reference planes were the scapular plane (xplane), and the y and z planes (which were orientated perpendicular to the scapular plane). The scapular plane was defined by the location of points C, M and I [15]. The scapular plane (analogous to a coronal plane) was designated the x plane. The yplane is orthogonal to the scapular plane and is orientated anteroposteriorly (analogous to the axial plane). The z plane is orthogonal to the scapular plane and superoinferiorly (analogous to the sagittal plane) (Fig. 5b). The orientation of the mesh perpendicular to the scapular plane (x plane) and parallel to the y and z planes served as the baseline position. Planes can be also defined by the current viewing perspective (viewing plane) (Fig. 6). The viewing planes were: perpendicular to the undersurface of the acromion (‘undersurface acromion viewing plane’); a view in which the acromion was viewed from posterolaterally with the mesh in a position at which the curved appearance of the junction of the scapular spine and the acromion was maximized (‘posterolateral acromion viewing plane’). The orientation of the mesh perpendicular to the y plane and parallel to the x and z planes created the ‘axial viewing plane’. The orientation of the mesh perpendicular to the z plane and parallel to the x and y planes created the ‘sagittal viewing plane’.

Once defined, planes (either reference planes or viewing planes) can be marked with a visible grid (Fig. 6b). The grid can be used as a tool to orientate the mesh because when the mesh is viewed perpendicular to the grid it will appear as a line (named here as a ‘gridline’) (Fig. 6c).

**Fig. 7:**
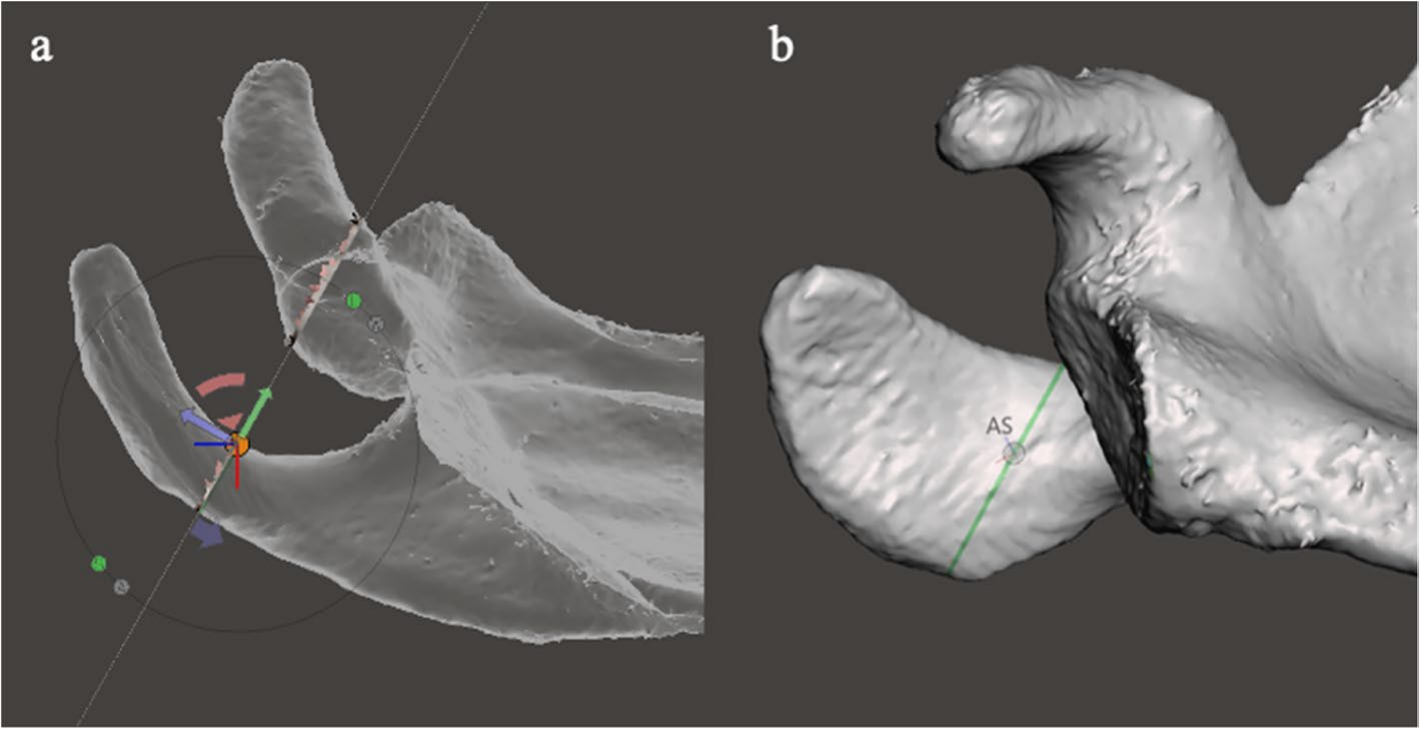
Illustration of the method of defining the acromiospinal (AS) point: **a** the posterolateral acromion view of the mesh is established, and a viewing plane and grid are inserted at the point where the scapular spine merges with the acromion. **B** The mesh is placed in the undersurface acromion view, while leaving the posterolateral acromion view grid (green line) in situ. The AS point is defined as that point on the posterolateral acromion viewing plane grid that is at the junction of the acromion and the spine, visible as the termination of a v-shaped prominence. The AS point is marked with a pivot

Planes, grids and gridlines were used to define other landmarks. These were the acromiospinal (AS) point and the spinoglenoid (SG) point and were used for the decomposition of the scapula into subparts. The AS point was defined as the junction of the acromion and the scapular spine (Fig. 7).

**Fig. 8:**
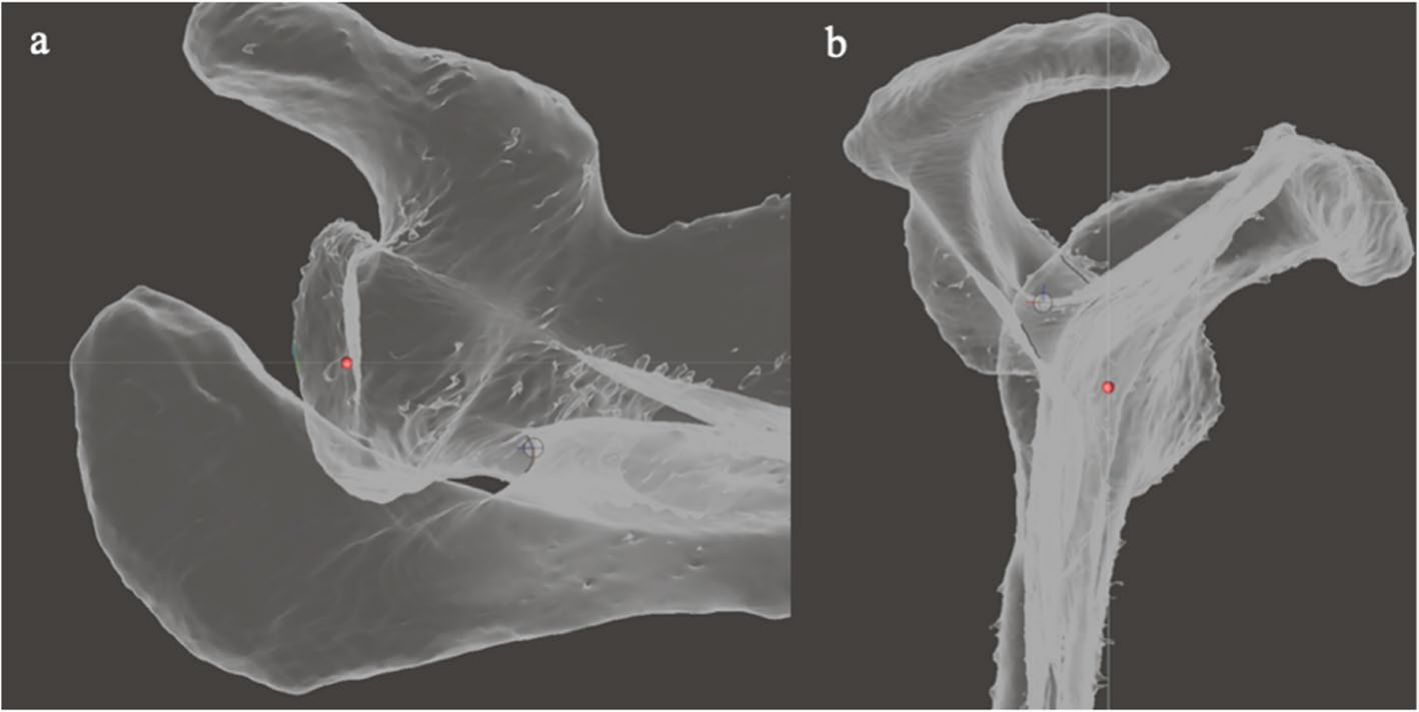
Illustration of the method of defining the spinoglenoid (SG) point. **a** In the axial viewing plane, a pivot is placed at the apex of the spino-glenoid notch. **b** The mesh is reorientated to the sagittal viewing plane, and the pivot is moved to the centre of the spine by moving it along the z axis

The SG point was defined as the junction of the scapular spine with the glenoid (Fig. 8).

### Measurements

**Fig. 9:**
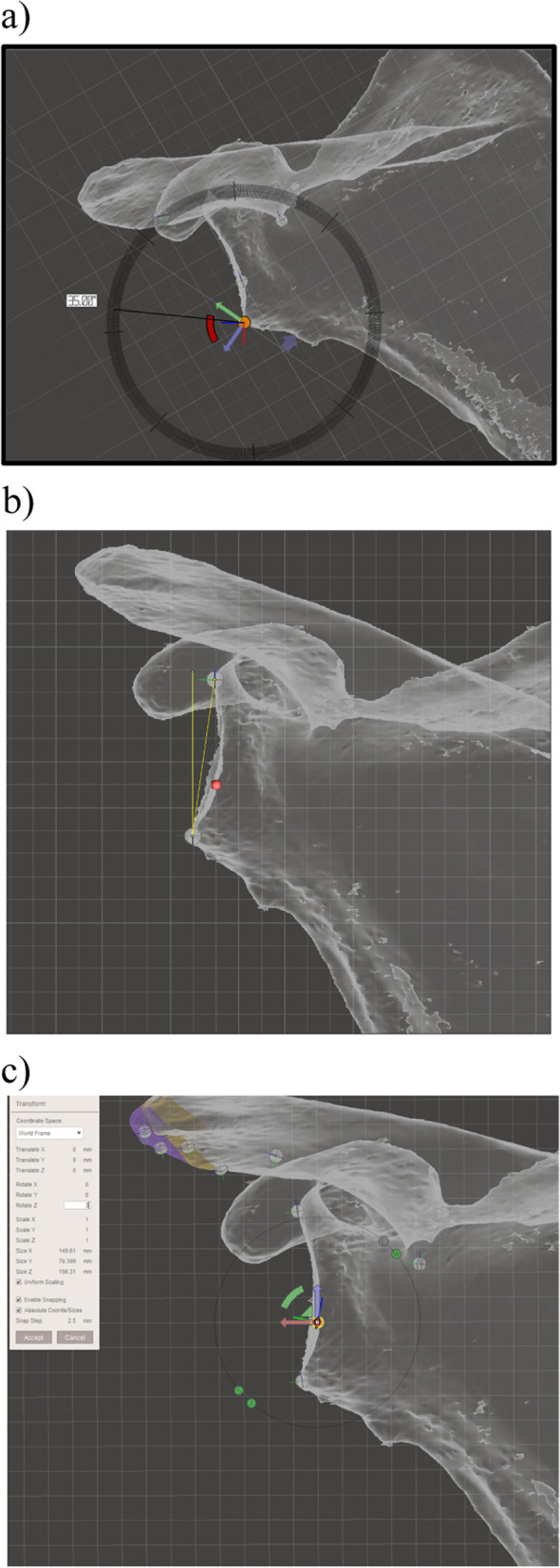
Illustration of the method used for: **a** CSA measurement: The mesh is placed in the baseline position a manipulated until a Suter-Henninger A1 view was achieved. A 2D angular measurement of the CSA is then performed; **b** Glenoid inclination: The mesh is manipulated to the baseline position; the glenoid inclination is the angle between a line running from the superior to the inferior margin of the glenoid and the z-axis; **c** Glenoid version: The mesh is placed in the baseline position. It is then rotated about the point C until there is optimal overlap between the anterior and posterior glenoid rims. The angle of rotation of the mesh required to achieve the A1 view defines the glenoid version (in this case 3°)

**Fig. 10:**
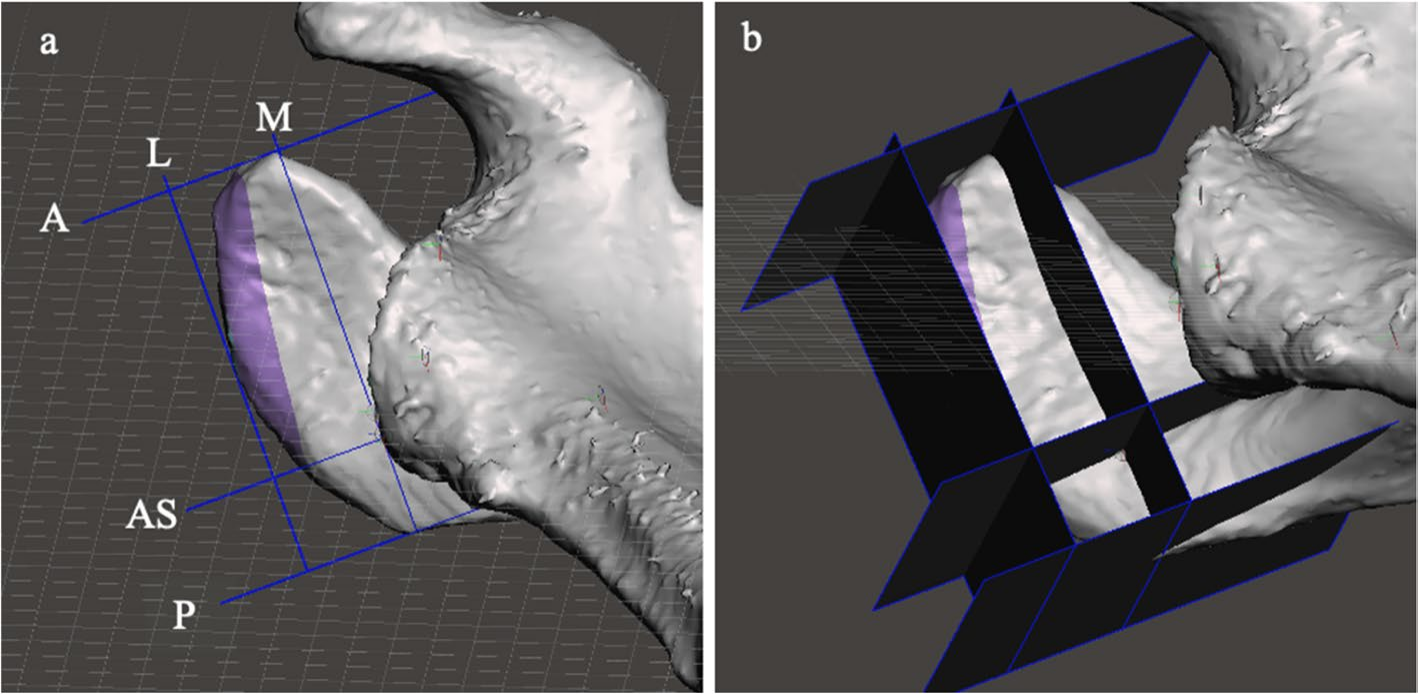
Illustration of the method of measuring acromial width, length and area: **a** The mesh is placed in the undersurface acromion viewing plane. The ‘lateral border acromial plane’ (running along a line of best fit along the lateral acromial border) and the ‘mid acromial plane’ (running parallel to the lateral border acromial plane and running along the longitudinal axis of the acromion through the acromiospinal point) defined the lateral (L) and medial (M) borders of an ‘acromial box’ and are marked. Orthogonal planes at the anterior- and posterior-most part of the acromion are inserted to define the anterior (A) and posterior (P) borders of the ‘acromial box’. A further plane that is parallel to the anterior and posterior borders of the acromial box and which intersected the AS point is inserted. This plane (AS) divided the ‘acromial box’ into an ‘anterior acromial box’ and a ‘posterior acromial box’. **b** when viewed obliquely the planes appear in 3D rather than as lines

**Fig. 11:**
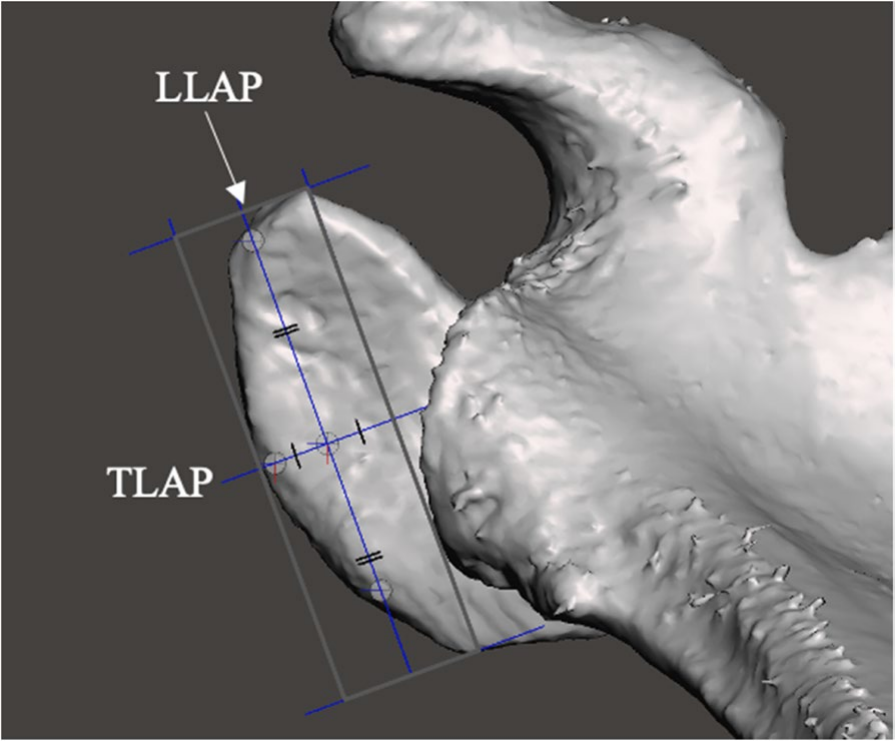
Illustration of the method used to define the acromial axes. Using the undersurface acromion viewing plane, a plane is inserted that bisects the acromial box longitudinally (the ‘longitudinal lateral acromial plane’[LLAP]). A second plane is inserted that bisected the acromial box transversely (the ‘transverse lateral acromial plane’[TLAP]). The junction of these planes with the bone along the inferior border of the acromion at their intersection, the most anterior, posterior and lateral points are marked with pivots. These pivots are connected to create the LLAP and TLAP

**Fig. 12:**
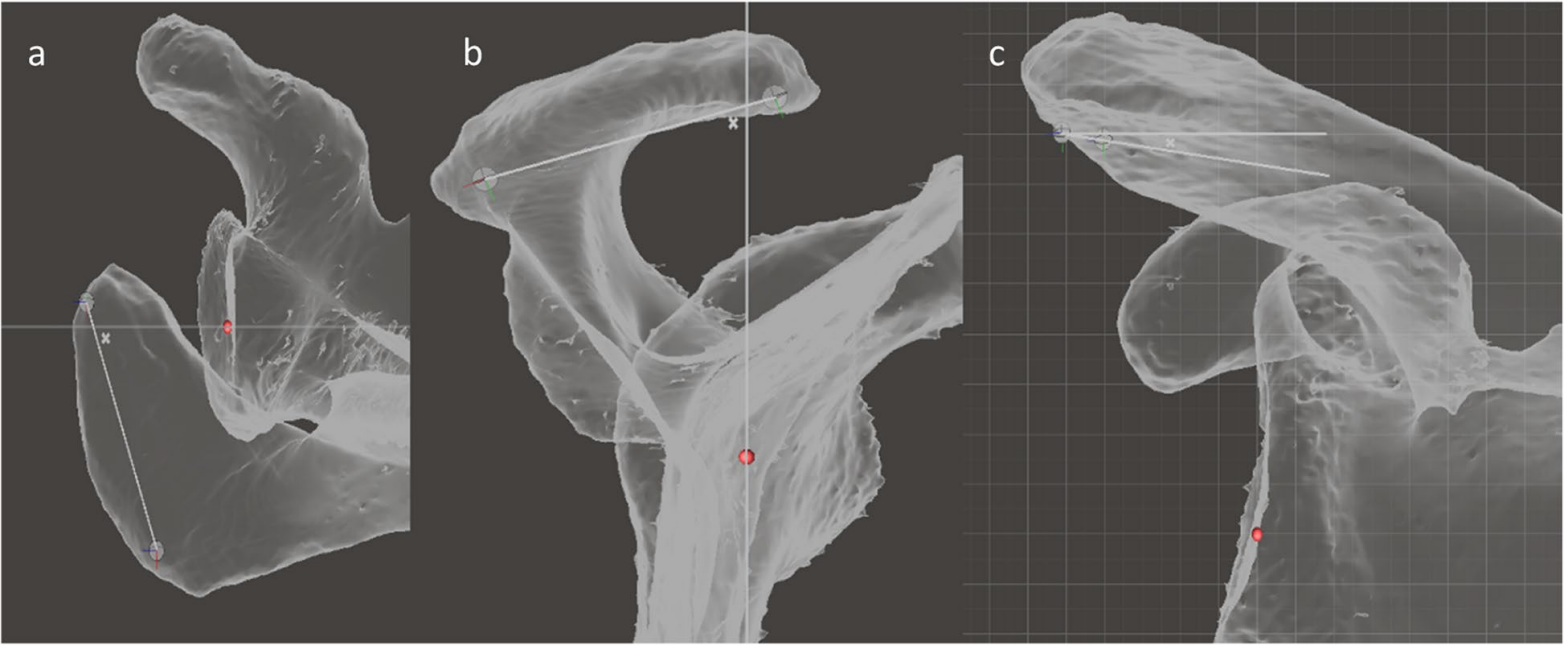
Assessment of the acromial orientation: **a** Axial plane angulation of the acromion (APAA): The mesh is positioned in the axial viewing plane and the angle between the longitudinal lateral acromial axis and the y axis is measured (angle labelled x); **b** Sagittal plane angulation of the acromion (SPAA): the mesh is positioned in the sagittal viewing plane and the angle between the longitudinal lateral acromial axis and the z axis is measured (angle labelled x); **c** Coronal plane angulation of the acromion (CPAA): the mesh is placed in the baseline position and the angle between the transverse lateral acromial axis and the x axis is measured (angle labelled x)

**Fig. 13:**
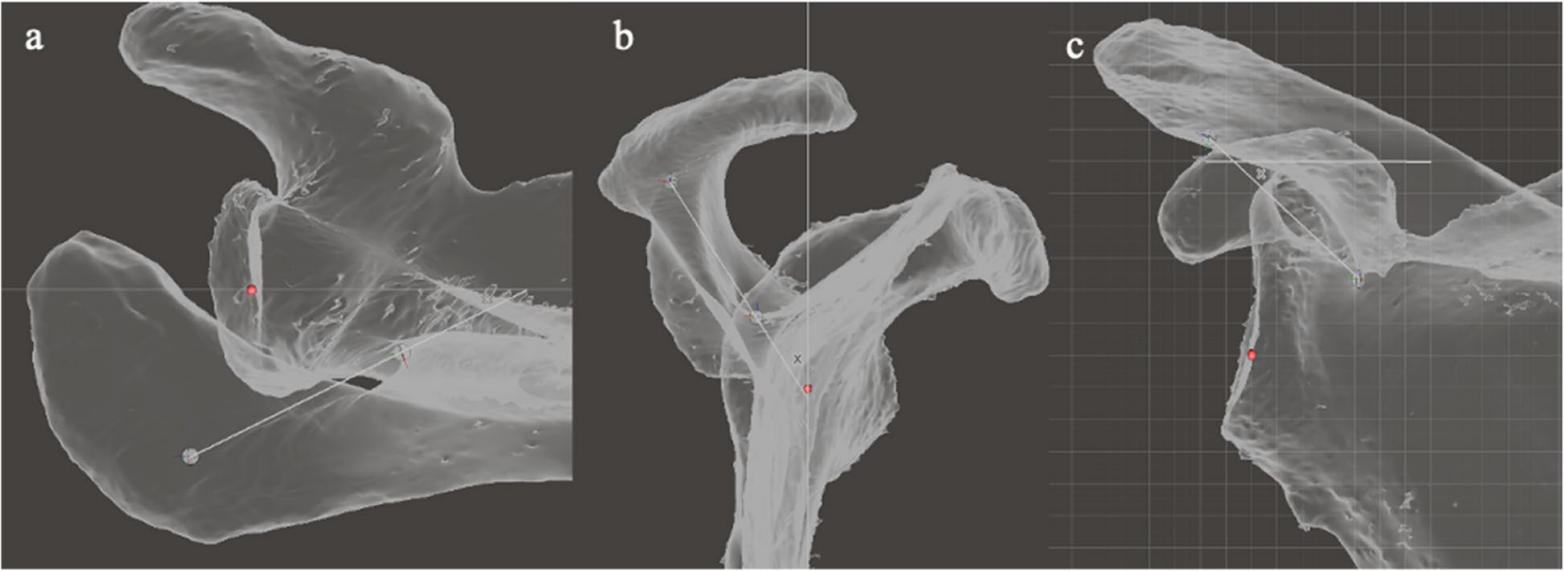
Assessment of the scapular spinal orientation: **a** Axial plane scapular spine angulation (APSSA): The mesh is positioned in the axial viewing plane and the angle between the scapular spinal axis and the y axis is measured **b**) Sagittal plane scapular spine angulation (SPSSA): the mesh is positioned in the sagittal viewing plane and the angle between the scapular spinal axis and the z axis is measured (labelled x). **c** Coronal plane scapular spine angulation (CPSSA): the mesh is placed in the baseline position and the angle between the scapular spinal axis and the x axis is measured (labelled x)

The CSA, GI, and GV were measured along with measures of acromial geometry (width, length, area), acromial orientation (CPAA, SPAA, APAA), scapular spine geometry (length), and scapular spine orientation (CPSSA, SPSSA, APSSA) (Figs. 9, 10, 11, 12 and 13).

Because the study population is a single cohort of patients with no comparison group, linear measurements were considered potential sources of bias because variations in geometric measurements could be due to patient biological sex and size rather than CSA. Therefore, a weighted value (wt) was calculated by dividing each linear measurement by the sum of CM, MI and CI. This generated the following weighted measurements: acromial box width wt, anterior acromial box length wt, posterior acromial box length wt, spinal height wt and spinoglenoid distance wt in the x, y and z planes.

Seventeen variables were considered to be possible explanatory variables for the measured CSA (the outcome variable) based on scapular anatomy were: GI; GV; acromial box width wt; anterior acromial box length wt; posterior acromial box length wt; anterior acromial box area; posterior acromial box area; CPAA; SPAA; APAA; spinal length wt; CPSSA; SPSSA; APSSA; spinoglenoid distance in the x plane wt; spinoglenoid distance in the y plane wt; spinoglenoid distance in the z plane wt.

### Statistical analysis

Analysis was performed using R (R Core Team, 2020, *R: A language and environment for statistical computing*, R Foundation for Statistical Computing, Vienna, Austria. URL https://www.R-project.org/, version 4.0.2) by a Statistician (PG-S).

The correlation of each of the explanatory variables with the CSA was assessed with the Pearson’s correlation coefficient. A multivariable linear regression prediction model with the normal assumption was selected as the CSA is a continuous variable. The model-building approach was leave-one-out cross-validation together with best subset selection. The average mean squared error (MSE) for models of each size was calculated, the smallest average value indicating the model of optimal size. The best model of that size (that with the smallest MSE) fitted to the full data set was adopted as the optimal prediction model. The variance inflation factor (VIF) was calculated to assess the degree of collinearity present. Collinear variables were removed one at a time from the model in separate model-building runs to determine the overall optimal model. The model validation procedure employed enables selection of a model with the best properties for prediction based on the optimal test error rate [23]. The assumptions of normal distribution and the constant variance of residuals were assessed for the initial full model and the final optimal model using diagnostic plots. The Akaike information criterion (AIC) and adjusted R-squared were calculated using drop one variable selection on the optimal prediction model to assess the importance of the explanatory variables in predicting the CSA.

## Results

Ninety-two CT scans were screened. Thirty-nine were excluded (3 with slice thickness > 1 mm; 11 gross glenoid or acromial deformity; 11 artefact; 14 incomplete capturing of the entire scapula). 53 CT scans were included. The mean CSA was 32.8^o^ (SD: 4.7). 14 cases had a CSA < 30°, 26 had a CSA between 30–35° and 13 had a CSA > 35°. The demographic data of the patients and their diagnoses are presented in Table 1. A summary of the descriptive results and the correlations of all the explanatory variables with the CSA is presented in Table 2.

**Table 1:** Demographic details and diagnoses of the included patients in the study

	CSA < 30 (*n* = 14)	CSA 30–35 (*n* = 26)	CSA > 35 (*n* = 13)
Mean Age: Yrs (SD)	48.9 (24.5)	52.9 (22.5)	59.2 (14.3)
Gender: Male: Female	10 (71.4%): 4 (28.6%)	16 (61.5%): 10 (38.5%)	6 (46.2%): 7 (53.8%)
Side Right:Left	6: 8	14:12	7:6
Diagnosis: N(%)			
Glenohumeral instability	6 (42.9%)	8 (30.8%)	2 (15.4%)
Fracture	4 (28.6%)	10 (38.5%)	9 (69.2%)
Glenohumeral OA	4 (28.6%)	7 (26.9%)	1 (7.7%)
Rotator cuff arthropathy	0 (0%)	1 (3.8%)	1 (7.7%)

**Table 2:** Summary of the descriptive results and the correlations of all the potential explanatory variables with the critical shoulder angle

Explanatory Variable	Mean [SD]	Pearson’s Correlation Coefficient
Glenoid inclination (^o^)	7.14 [4.10]	0.535
Glenoid version (^o^)	-4.75[-5.16]	-0.121
Acromial Box Width wt	0.14 [0.04]	0.249
Anterior Acromial Box Length wt	0.30 [0.04]	-0.006
Posterior Acromial Box Length wt	0.12 [0.02]	0.101
Anterior Acromial Box Area	0.77 [0.10]	-0.030
Posterior Acromial Box Area	0.62 [0.11]	-0.050
Axial plane angulation of the acromion (^o^)	65.12 [9.80]	-0.102
Sagittal plane angulation of the acromion (^o^)	61.23 [9.39]	-0.426
Coronal plane angulation of the acromion (^o^)	7.16 [15.42]	-0.502
Spinal Length Wt	0.29 [0.04]	-0.259
Coronal plane scapula spine angulation (^o^)	37.96 [5.94]	-0.172
Axial plane scapula spine angulation (^o^)	35.61 [7.23]	0.205
Sagittal plane scapula spine angulation (^o^)	42.66[9.37]	0.283
Spinoglenoid distance x plane wt	0.17 [0.02]	-0.065
Spinoglenoid distance y plane wt	0.07 [0.01]	0.146
Spinoglenoid distance z plane wt	0.07 [0.02]	-0.352

Three explanatory variables demonstrated high collinearity, based on large VIF’s: APSSA, CPSSA and SPSSA. Based on scatter plots and VIF’s, all the candidate predictor variables were sufficiently linearly related to CSA to justify a linear model.

The cross-validation assessment demonstrated that a model with 10 explanatory variables had the lowest mean square error (MSE) and highest adjusted R-squared (0.67) in the prediction of the CSA. The assumptions of normal distribution and constant variance of residuals were judged to be satisfied. No variable in the data contained missing values or extreme values. The best combination of 10 explanatory variables had an MSE of 7.33 and an adjusted R2 of 0.67. The estimate of the coefficients for the best combination of explanatory variables and the AIC and adjusted R-squared for the drop one validation are shown in Table 3. From that table, the most important predictors were CPAA and GI.

**Table 3:** Estimates of the coefficients in the optimal model and results of dropping each explanatory variable separately (AIC and adjusted R-squared) from the explanatory variables in the optimal model for critical shoulder angle (*p*-value is not a criterion according to which the optimal model was chosen)

	Estimate of the coefficients	95% confidence interval lower bound	95% confidence interval upper bound	*p* value	Model AIC on removing variable	Model Adjusted R2 on removing variable
Glenoid inclination	0.625	0.384	0.866	< 0.001	139.9	0.467
Glenoid version	-0.177	-0.347	-0.008	0.041	118.6	0.641
Anterior Acromial Box Length Wt	-29.758	-57.372	-2.144	0.035	118.9	0.641
Posterior Acromial Box Length Wt	63.756	21.682	105.829	0.004	123.9	0.606
Sagittal plane angulation of the acromion	0.186	0.043	0.330	0.012	121.3	0.625
Coronal plane angulation of the acromion	-0.202	-0.263	-0.140	< 0.001	151.3	0.339
Sagittal plane scapular spine angulation	0.230	0.099	0.362	0.001	127.0	0.582
Spinoglenoid distance x plane wt	-58.680	-106.351	-11.010	0.017	120.5	0.630
Spinoglenoid distance y plane wt	-40.408	-93.252	12.435	0.130	116.2	0.659
Spinoglenoid distance—z plane Wt	-63.583	-115.214	-11.951	0.017	120.5	0.630

## Discussion

The results of this study demonstrated that a model comprising 10 explanatory morphological variables was the most accurate in predicting the CSA value. Therefore, the relationship between the scapular morphology and the CSA value is more complex than the concept of it being dictated solely by the GI and the acromial ‘width’; instead, glenoid, scapular spine and acromial factors are all relevant.

Among the combination of explanatory variables in the best 10-factor model, CPAA and GI are the most important based on their distribution, the estimate of the coefficients and the loss in predictive power of the model if they were removed. Although the remaining 8 factors in the best 10-factor model had additional predictive value, their individual influence seems less strong. This statistical approach, which incorporated different modelling methods and a number of criteria for judging model fit to determine a "consensus" model, was selected in light of the small size of the data set relative to the number of potential predictors. This meant that there was no single statistical method that could be relied upon to determine a single best set of active predictors of CSA. That the best 10 factor predictive model had an R-squared value of 0.67 raises the possibility that other morphological variations that were not considered here may be influential, or that the measurement technique lacked the ability to precisely quantify the effect of those that were measured.

Along with its retrospective design and the lack of comparative cohorts, the major limitation of the present study is that the population was heterogenous and included all patients with any symptomatic shoulder pathology that underwent investigation with CT. Therefore, the results of this study may not reflect those of a population of patients with RC tears and primary GHOA in which a relationship with variances in CSA has been established. Therefore, further studies are required to assess this. A previous 2D study identified that patients with massive RC tears had a more ‘downward tilting’ ‘acromial roof’ that was more externally rotated and wider than in those with concentric GHOA [6]. The importance of the CPAA (most analogous to ‘coronal tilt’ of the ‘acromial roof’) in the current study is consistent with those previous findings. Conversely, APAA (most analogous to ‘axial tilt’ of the ‘acromial roof’) and acromial box width (most analogous to ‘acromial roof’ ‘width’) were not identified in the best predictive model in the present study though they were considered as potential explanatory variables.

Other than the study population, the previously described advantages of the 3D method of the assessment of scapular morphological variances that uses the scapular plane and the scapula axis compared to a 2D methodology that uses the glenoid as a reference axis could explain the difference in results. The measurement of the geometry and orientation of the lateral part of the acromion in this study, rather than the entire acromion, may also explain the difference in results; this method being considered advantageous because it reflects the importance of the deltoid origin at the lateral acromion in the biomechanical concept of the CSA. Finally, the use of weighted longitudinal measurements to eliminate the influence of body habitus in the current study, as well as the ability to statistically adjust for all other morphological factors may also explain the differences.

A major interest in the measurement of the CSA is that surgical modification of scapular morphology may be a strategy to prevent the development of RC tears or the prevention of failure to heal after RC repair [1, 17, 24, 25, 29]. The population of the current study does not allow any conclusions to be drawn about which of the predictive factors that are associated with variances in the CSA may be used to define the ‘high risk scapula’ for the development of RC tears as opposed to a high CSA value per se. This would require the current study to be replicated in a population of patients with RC tears. However, an assessment of scapular morphology based on the explanatory variables used in this study may be more relevant than the measurement of the CSA. A radiographic method would be advantageous because it does not require any additional imaging that may be considered ‘non-standard’. But of the 10 explanatory variables in the best predictive model only the measurement of GI and CPAA would be possible using a 2D assessment of plain radiographs. While the 2D plain radiographic assessment of GI could be an accurate measure of this scapular variance (if the imaging plane was in the glenoid plane and a suitable reference axis could be designated), it is important to recognise that the 2D measurement of the CPAA would not truly reflect this variance as the acromion does not lie in the same plane as the glenoid. Instead, the 2D measured CPAA value would be a composite measure which reflects a number of scapular morphological variances. For this reason, if the CPAA was measured in a 2D assessment it is recommended that it be suffixed -m to identify this issue as a potential confounder for its value.

A 3D assessment of scapular morphology would be required to assess each of the best set of active predictors of CSA that were identified in this study, necessitating the use of CT as an investigation for all patients with RC tears rather than only in those patients with massive RC tears or cuff tear arthropathy that are under consideration for reverse shoulder arthroplasty. However, the subsequent methodology used in this study is too time-consuming for use in routine clinical practice and the additional measurement of the 8 other explanatory variables may not be justified considering their less strong influence on the CSA.

From a practical standpoint, a lateral acromioplasty is the simplest surgical method of modifying some of the explanatory variables identified in this study. Should the results of this study be replicated in a population of patients with RC tears and further biomechanical studies demonstrate their relevance, a multiplanar lateral acromioplasty could be considered to address all the acromial factors in the best predictive model for the CSA, aiming to diminish the CPAA, acromial length and SPAA (but not acromial width).

## Conclusions

The relationship between the scapular morphology and the CSA value is complex and includes glenoid, scapular spine and acromial factors. The coronal plane angulation of the acromion and the glenoid inclination are the most important predictive variables. A further investigation in a closely defined cohort with rotator cuff tears is required before drawing any clinical conclusions about the role of surgical modification of scapular morphology.

## Data Availability

The datasets generated during and/or analysed during the current study are available from the corresponding author on reasonable request.

## Abbreviations

AIC: Akaike information criterion
APAA: Axial plane angulation of the acromion
APSSA: Axial plane scapular spine angulation
AS point: Acromiospinal point
CAP: Critical acromial point
CPAA: Coronal plane angulation of the acromion
CPSSA: Coronal plane scapular spine angulation
CSA: Critical shoulder angle
CT: Computerised tomography
DICOM: Digital imaging and communications in medicine
GHOA: Glenohumeral osteoarthritis
GI: Glenoid inclination
GV: Glenoid version
LLAA: Longitudonal lateral acromial axis
MSE: Mean squared error
MPR: Multiplanar reconstruction
RC: Rotator cuff
SG point: Spinoglenoid point
SPAA: Sagittal plane angulation of the acromion
SPSSA: Sagittal plane scapular spine angulation
TLAA: Transverse lateral acromial axis
VIF: Variance inflation factor
